# Validation of the IWATE Criteria in Robotic-Assisted Liver Resections

**DOI:** 10.3390/jcm13092697

**Published:** 2024-05-03

**Authors:** Sophia A. Lamberty, Jens Peter Hoelzen, Shadi Katou, Felix Becker, Mazen A. Juratli, Andreas Andreou, M. Haluk Morgül, Andreas Pascher, Benjamin Strücker

**Affiliations:** Department of General, Visceral and Transplant Surgery, University Hospital Muenster, 48149 Muenster, Germany; sophia.lamberty@ukmuenster.de (S.A.L.); shadi.katou@ukmuenster.de (S.K.); felix.becker@ukmuenster.de (F.B.); mazen.juratli@ukmuenster.de (M.A.J.); andreas.andreou@ukmuenster.de (A.A.); haluk.morguel@ukmuenster.de (M.H.M.); andreas.pascher@ukmuenster.de (A.P.); benjamin.struecker@ukmuenster.de (B.S.)

**Keywords:** IWATE criteria, liver resection, minimally invasive surgery, robotic surgery

## Abstract

**Background/Objectives**: The IWATE criteria are well-established as a helpful tool to preoperatively estimate the difficulty and perioperative outcome of laparoscopic liver resections. We evaluated the relationship between the IWATE criteria and the perioperative outcomes in robotic-assisted liver resections (RARLs). **Methods**: We retrospectively analyzed the data of 58 patients who underwent robotic-assisted liver surgery at our center between July 2019 and April 2023. The operative difficulty of every patient was graded according to the IWATE criteria and compared to the perioperative outcome. **Results**: The median operation time was 236.5 min (range 37–671 min), and the median length of stay was 6 days (range 3–37 min). The majority had no complications (65.5%; *n* = 38), 18 (31.0%) patients suffered from mild complications (CD ≤ 3A) and 2 patients (3.4%) suffered from relevant complications (CD ≥ 3B). We observed no deaths within 30 postoperative days. The surgery time, postoperative ICU stay and perioperative blood transfusions increased significantly with a higher difficulty level (*p* = < 0.001; *p* < 0.001; *p* = 0.016). The length of stay, conversion to open surgery (*n* = 2) and complication rate were not significantly linked to the resulting IWATE group. **Conclusions**: The IWATE criteria can be implemented in robotic-assisted liver surgery and can be helpful in preoperatively estimating the difficulty of robotic liver resections. Whether there is a “robotic effect” in minimally invasive liver resections has to be further clarified. The IWATE criteria can help to develop curricula for robotic training.

## 1. Introduction

For most indications, minimally invasive liver surgery has shown superior outcomes in comparison to open surgical procedures [1,2,3,4]. Several studies have demonstrated the advantages of laparoscopic liver resections (LLRS) as a safe surgical procedure [4,5]. LLR is associated with less postoperative pain and perioperative complications, a shorter hospital stay and a similar oncologic outcome compared to open liver surgery [1,2,3]. However, LLR is a highly demanding and technically complex surgical procedure with a steep learning curve [6].

To ensure the safe applicability of LLR in patients and avoid negative outcomes, the IWATE criteria were discussed at the 2nd International Consensus Conference on Laparoscopic Liver Resection (ICCLLR) 2014, held in Morioka, Japan and published afterward [7]. The IWATE criteria consist of six preoperatively collected parameters, resulting in four difficulty levels for LLRs. Each parameter is scored differently, resulting in a total score related to one of the four difficulty groups [8]. The IWATE criteria represent a further development of the Difficulty Scoring System (DSS) by Ban et al. which originally used three difficulty levels and was the first known DSS for LLR [9]. Major changes in the DSS were made due to a shift to more complex liver resections, with the use of hand-assisted laparoscopic surgery (HALS), resections of the first segment and differentiation between segments IVa and IVb added [7].

Parallel to the establishment of LLR, the implementation of robotic-assisted liver resections (RALRs) has gained more attention and recognition in recent years [10,11,12]. RALR provides a greater range of motion, high-resolution 3D visualization, elimination of the surgeon’s tremor and improvement of the surgeon’s ergonomics [13,14,15]. This results in comparable postoperative outcomes but lower conversion rates and less intraoperative blood loss in comparison to LLRs [13,16,17,18,19,20,21,22,23].

DSS can be helpful for surgeons when introducing new surgical techniques and can ensure their feasibility in a manner that is safe for patients. Prediction of intra- and postoperative outcomes using the IWATE criteria in LLRs has been validated in several studies [8,24,25,26]. Therefore, the IWATE criteria are considered a useful tool to select patients for LLRs according to the surgeon’s experience and skill level [8,24]. However, the applicability of the IWATE criteria in RALR has not yet been adequately established. The aim of this study was to investigate the applicability of the IWATE criteria in RALR by comparing their categorization with the perioperative outcome. Furthermore, we investigated whether the robotic approach might alter the categorization of the IWATE criteria in recognition of a potentially alleviating effect (i.e., robotic effect) on the perioperative outcome [27]. In our study, we retrospectively categorized all patients according to different levels of operating difficulty using the IWATE score and subsequently compared those categorizations with the perioperative outcomes.

## 2. Materials and Methods

### 2.1. Study Design and Population

This retrospective, single-center study was carried out at the University Hospital of Münster, Germany and approval was given by the local ethics committee (ID: ID2019-636-f-S). All patients (*n* = 58) who underwent a robotic-assisted liver resection between July 2019 and April 2023 were included in this trial. Indications for resections were primary hepatobiliary malignancies including hepatocellular carcinoma (HCC), intrahepatic cholangiocarcinoma (ICC), gallbladder carcinoma (GBC), benign liver tumors and metastatic liver tumors. We excluded patients undergoing liver cyst marsupialization and concomitant extrahepatic procedures, except for cholecystectomy, hernia repair and partial diaphragmatic resection. The decision of surgery was made by our local interdisciplinary tumor board. Patient data were compiled from in-clinic information systems and randomized for further analysis.

### 2.2. Collected Data and Definitions

Patients’ demographic data including age, sex, body mass index (BMI), American Society of Anesthesiologists (ASA) score, abuse of alcohol and smoking history, chronic hepatitis, previous abdominal surgery and chemotherapy and the presence of extrahepatic metastases were assessed. Pre-existing moderate or severe liver cirrhosis, steatosis and non-alcoholic fatty liver disease (NAFLD) were recorded. Furthermore, comorbidities like pulmonary and cardiac diseases, arterial hypertension, diabetes and renal insufficiency were gathered and analyzed for potential bias. Preoperative administration of chemotherapy included all types of chemotherapies prior to the robotic-assisted liver resection and was recorded for potential effects on liver quality and outcome.

Perioperatively collected data included surgery time, conversion rate, intensive care unit (ICU) stay, reoperation rate and length of stay (LOS). The operation time was defined as the time from the first skin incision to the final skin closure. Concomitant procedures during the operation rarely involved radiofrequency ablation (RFA) or lymphadenectomy (LAD). Perioperative blood transfusions included all types of red blood cell, platelet, plasma and granulocyte transfusions. Prior abdominal surgery was defined as any previous surgery that involved the upper or lower abdomen intraperitoneally. Postoperative complications were graded using the Clavien–Dindo Classification (CD) [28]. Based on the Clavien–Dindo Classification, a complication rate was developed including three groups: no complications (CD = 0), low (CD ≤ 3A) and relevant (CD ≥ 3B). Liver segments were identified based on Couinaud’s segmental anatomical classification [29]. Anatomical resections were defined based on the Brisbane 2000 Terminology of Liver Anatomy and Resections [30]. Short-term perioperative outcomes such as length of stay, 30-day readmissions, morbidity and mortality were assessed.

### 2.3. IWATE Criteria

The IWATE criteria consist of six preoperative factors used to predict the difficulty of liver resections. Tumor location (according to the different liver segments), tumor size (≤30 or ≥30 mm), liver function (Child–Pugh score A or B), extent of liver resection (partial resection, left lateral sectionectomy, segmentectomy, sectionectomy or more), proximity to major vessels (if the tumor is within 1 cm of the major hepatic veins, inferior vena cava or main branches of portal pedicle) and the use of hand-assisted laparoscopic surgery (HALS)/hybrid are combined into a single score. Preoperative imaging (CT and/or MRI and/or PET–CT scans) was used to determine tumor location, tumor size and proximity to major vessels. If multiple liver segments were affected equally by the tumor, the segment with the higher difficulty level was chosen. The IWATE criteria were used to categorize the following groups of operative difficulty levels: Low (score 1–3), Intermediate (score 4–6), Advanced (score 7–9) and Expert (score 10–12).

### 2.4. Operative Technique

All robotic-assisted procedures were performed using the da Vinci Surgical Xi System™ (Intuitive Surgical, Inc., Sunnyvale, CA, USA). A four-trocar technique with one or two assistance trocars was used in each case. Parenchymal dissection was performed with a combination of hook, SynchroSeal (Intuitive Surgical, Inc., Sunnyvale, CA, USA) and bipolar fenestrated forceps. For vascular control, Grena™ clips (Grena, London, UK) were used. TilePro (Intuitive Surgical, Inc., Sunnyvale, CA, USA) technology was used for intraoperative sonographic control, in which the sonographic image is superimposed directly onto the surgeon’s robotic field of view. In major procedures, the hepatoduodenal ligament was looped for temporary occlusion of the liver hilum. However, the Pringle maneuver was performed rarely. In individual cases, ICG was administered via i.v. one day before resection for better visualization of the tumor. No hybrid procedure was performed.

### 2.5. Statistical Analysis

Categorical variables were expressed as numerical figures and percentages and analyzed using Fisher’s exact test as appropriate. Continuous variables were expressed as medians and means and were assumed to be non-normally distributed. Comparisons among the groups were carried out by using the Kruskal–Wallis test. To investigate the association between the IWATE criteria and the perioperative outcome, we performed a correlation using Spearman’s correlation coefficient. A *p*-value < 0.05 (2-tailed) was considered statistically significant. We performed statistical analysis using SPSS (VIII.XX, IBM, Armonk, New York, NY, USA).

## 3. Results

### 3.1. Baseline Characteristics

**Figure 1:**
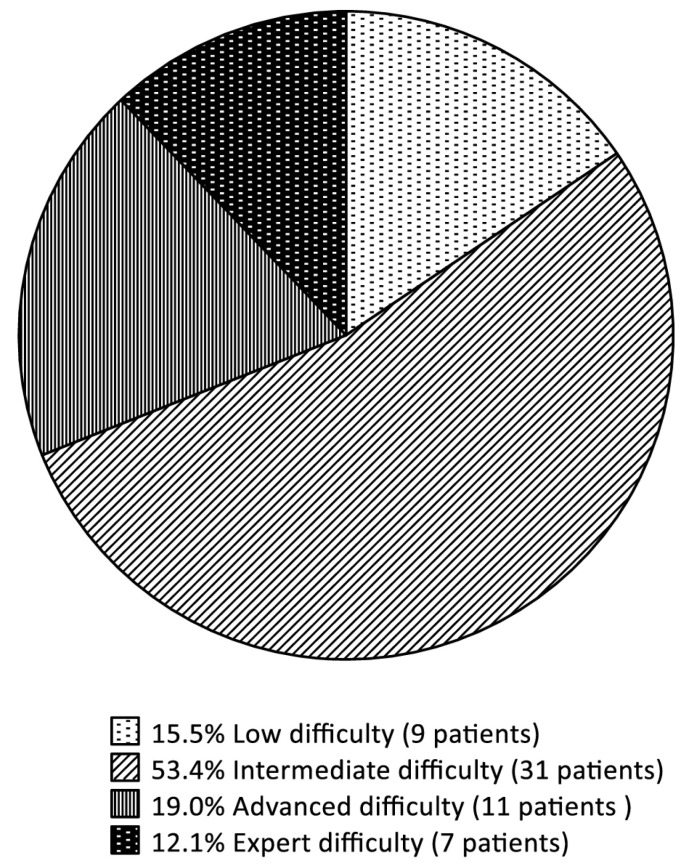
Pie chart displaying all patients who underwent robotic-assisted liver surgery, graded according to the IWATE criteria.

We performed 58 robotic-assisted liver resections at our institution between July 2019 and April 2023. According to the IWATE criteria, 9 patients (15.5%) were retrospectively categorized as Low, 31 (53.4%) as Intermediate, 11 (19.0%) as Advanced and 7 (12.1%) as Expert difficulty (Figure 1).

The median age of the population was 56 years (range 21–84 years) and female patients represented 51.7% of the sample. The most frequent indications for robotic-assisted liver surgery were colorectal liver metastasis (*n* = 12) and HCC (*n* = 11). Patients’ baseline characteristics are summarized in Table 1 and indications for resection in Table 2. There were no significant differences between the four levels of difficulty regarding the patients’ baseline characteristics. Some patients underwent chemotherapy prior to the operation due to malignancy (19.0%; *n* = 11), while 8.6% (*n* = 5) of patients suffered from extrahepatic metastases. In total, 60.3% (*n* = 58) of patients had undergone previous abdominal surgery including laparotomy or laparoscopic surgery.

### 3.2. IWATE Criteria and Perioperative Outcome

**Figure 2:**
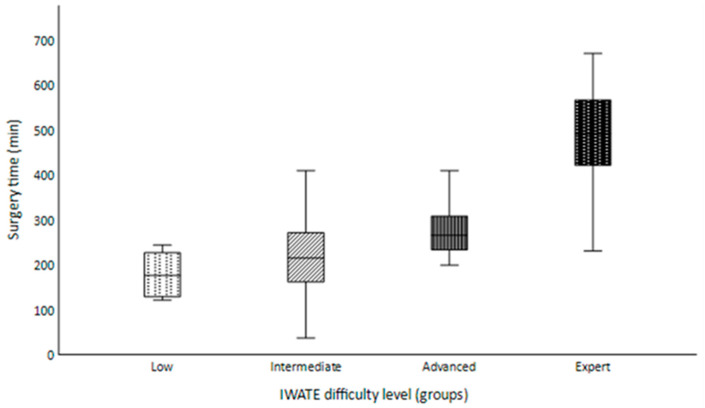
Box plot chart presenting the surgery time (minutes) in relation to the four difficulty levels.

Characteristics graded with the IWATE criteria are listed in Table 3, and the operative characteristics and perioperative outcomes are presented in Table 4. The distribution of tumor location and size, the extent of liver resection and proximity to major vessels within the four difficulty levels were congruent with the IWATE criteria. The majority of lesions had a size greater than 30 mm (62.1%; *n* = 36) and were located in liver segment III (19.0%; *n* = 11). There was only one patient with a liver function of Child–Pugh B in the Intermediate difficulty group, while all other patients were graded as Child–Pugh A or less (98.3%; *n* = 57). Proximity to major vessels was found in 60.3% (*n* = 35) of the patients. We mostly performed parenchymal-sparing resections (39.7%; *n* = 23) and no hybrid or hand-assisted laparoscopic surgery (HALS) was performed. The median surgery time was 236.5 min (range 37–671 min) and increased significantly at higher difficulty levels (Figure 2; *p* < 0.001). We observed the highest median operative time of 492 min (range 231–671 min) in the Expert difficulty group and the lowest median operative time of 176 min (range 121–244 min) in the Low difficulty group.

**Figure 3:**
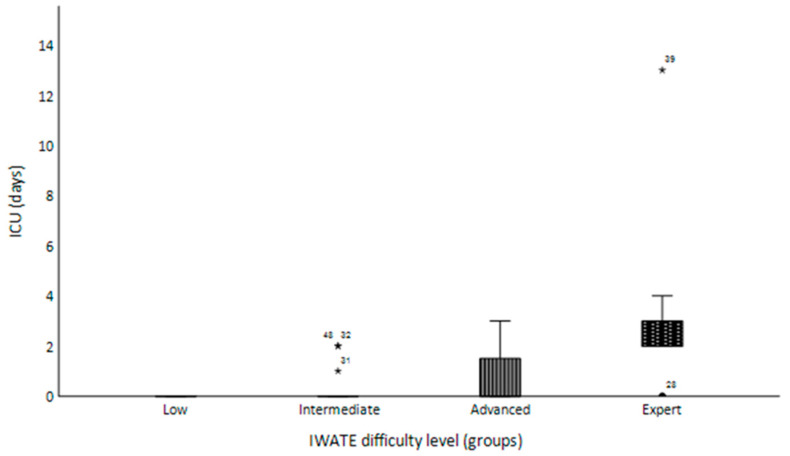
Box plot chart presenting the length of the ICU stay (days) in relation to the four difficulty levels. Statistical outliers and extreme outliers of the respective variable are displayed by asterisks in the box plot.

In the Advanced and Expert difficulty groups, two conversions to open surgery occurred due to impaired liver parenchyma in liver cirrhosis (Advanced difficulty group) and intraoperative hemorrhage with packing of the remnant liver in a case of a giant liver hemangioma (Expert difficulty group). However, the conversion rate showed no significant correlation with the IWATE criteria (*p* = 0.152). The Pringle maneuver was used most frequently in the Advanced (*n* = 2; 18.2%) and Expert difficulty levels (*n* = 3; 42.9%), with mean durations of 5.7 min (5.7 ± 12.8 min) and 20.0 min (20.0 ± 31.8 min), respectively. The use and duration of hilar occlusion differed significantly across the four difficulty levels (*p* = 0.016; *p* = 0.010). Postoperatively, 12 patients (20.7%) were transferred to the ICU with a mean length of stay of 0.7 days (0.7 ± 1.9). In the Expert difficulty group, 85.7% of patients (*n* = 6) had to stay in the ICU, with a mean length of stay of 3.6 days (3.6 ± 4.3 days), and no patients scored as the Low difficulty level were transferred to the ICU. The admission to and length of stay in the ICU significantly differed between the difficulty levels (Figure 3; *p* < 0.001).

**Figure 4:**
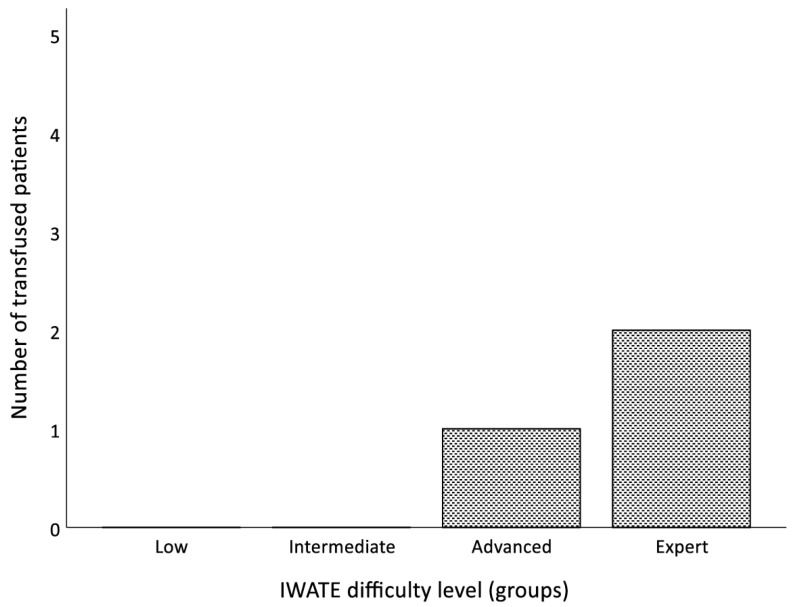
Bar chart displaying the perioperative blood transfusions (*n*) in relation to the four difficulty levels.

**Figure 5:**
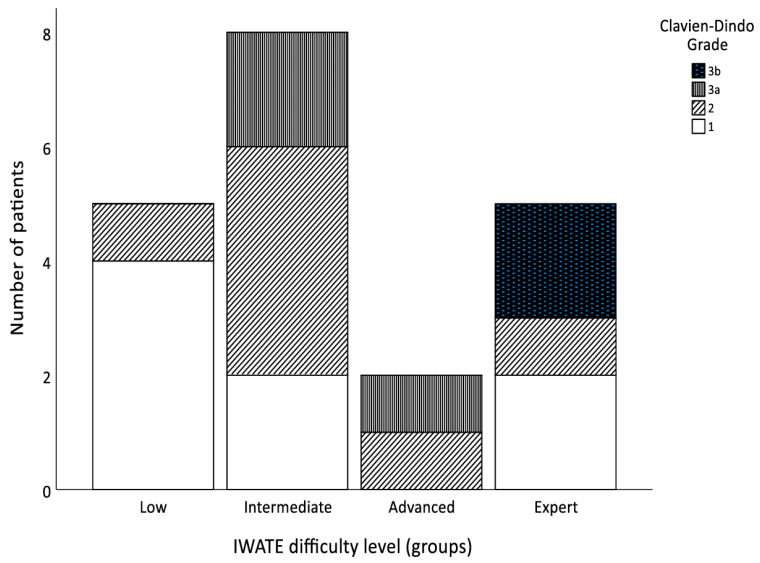
Bar chart displaying the Clavien–Dindo grade in relation to the four difficulty levels.

In total, three patients (5.2%) required blood transfusions, reaching statistical significance (*p* = 0.023) within the four groups (Figure 4). Of the seven robotic-assisted liver resections categorized as Expert level, 28.6% (*n* = 2) received blood transfusions perioperatively. Margin-free resection (R0) was achieved in 98.3% (*n* = 57) of the cases. Only one patient in the Expert difficulty group (giant liver hemangioma) required a reoperation after conversion due to intraoperative hemorrhage with packing of the remnant liver. Most patients had no complications (65.5%; *n* = 38) within 30 days postoperatively. Mild complications were graded as a Clavien–Dindo Classification ≤ 3A and occurred in 31% of the patients (*n* = 18). Postoperative complications according to the Clavien–Dindo Classification correlated significantly in the different groups (Figure 5; *p* = 0.014 and *p* = 0.018). The majority (28.6%; *n* = 2) of relevant complications (Clavien–Dindo Classification ≥ 3B) occurred after Expert-level resections. The most frequent complications postoperatively (15.5%; *n* = 9) were pulmonary (pneumonia, pleural effusions). However, non-surgical problems seemed to be significantly different between the four levels of difficulty (*p* = 0.013). Within 30 days postoperatively, four patients (6.9%) had to be readmitted to the hospital mainly because of intraabdominal seromas (*n* = 3) and one wound-healing disorder after conversion. The median length of stay was 6 days (range 3–37 days) and there were no deaths within 30 days postoperatively. There was no statistically significant difference in LOS within the four groups (*p* = 0.192).

According to Spearman’s correlation coefficient, the IWATE criteria correlated strongly with the surgery time (Spearman’s *p* = 0.591; *p* < 0.001) and the postoperative ICU stay (Spearman’s *p* = 0.542; *p* < 0.001). Also, the use and duration of the Pringle maneuver were moderately correlated with the IWATE criteria (Spearman’s *p* = 0.387; *p* = 0.003). The frequency of blood transfusions increased as the difficulty of the surgery progressed (*p* = 0.016; x^2^ test for trend). However, the IWATE criteria were not significantly linked to the conversion rate (*p* = 0.176; x^2^; test for trend) or LOS (Spearman’s *p* = 0.048; *p* = 0.720). Although the complication rates differed significantly between the four difficulty levels, the IWATE criteria were not associated with an increase in the postoperative complication rate (Spearman’s *p* = 0.046; *p* = 0.732).

## 4. Discussion

The IWATE criteria are well-established in LLRs and considered a helpful tool for preoperative risk stratification [8,24,25,26]. Although several studies have been published, the validity of the IWATE criteria in RALR still remains unclear [27,31,32,33]. In our study, we found that the IWATE criteria were strongly associated with surgery time and the postoperative ICU stay (*p* < 0.001). Moreover, the use and duration of Pringle’s maneuver and the need for perioperative blood transfusions were significantly higher with an increasing difficulty level (*p* = 0.003; *p* = 0.016). Contrary to our expectations, the IWATE criteria failed to significantly predict the conversion rate, postoperative complications or LOS.

Our findings differ compared to the results of other studies applying the IWATE criteria in LLR. Krenzien et al. showed a strong association between the IWATE criteria’s predictions of intraoperative surgical difficulty and subsequent data on postoperative morbidity and LOS in LLR for HCC patients [8]. Their conclusion was to recommend using the IWATE criteria as a predictor in LLR and in order to build a curriculum for upcoming surgeons. Another study, by Tanaka et al., published similar results after applying the IWATE criteria in LLR in high-volume centers in Japan and France [24]. Their conclusion was that the IWATE criteria could predict the intra- and postoperative outcomes of LLR. Moreover, the IWATE criteria were significantly linked with surgery time, EBL, postoperative complications, liver failure and in-hospital deaths.

While the applicability of the IWATE criteria has been clearly demonstrated for LLR, results regarding their application in RALR are rather inconsistent [8,24,25,26,27,31,32,33]. Labadie et al. applied the IWATE criteria in RALR and obtained similar results as Krenzien et al. and Tanaka et al. [31]. In their study, a higher IWATE difficulty level was associated with an increase in estimated blood loss (EBL), LOS and surgery time. However, these results differed significantly compared to the results of Luberice et al., who prospectively applied the IWATE criteria in patients undergoing RALR [27]. Their results showed that EBL and surgery time increased with a higher difficulty level; however, the postoperative outcome was irrespective of the IWATE criteria. They concluded that the robotic approach might have a mitigating effect on the postoperative outcome. In our study, we obtained similar results as Luberice et al., meaning that we cannot eliminate a “robotic effect”. Likewise, a recently published study by Steinkraus et al. showed the ability of the IWATE criteria to predict the difficulty of surgery and postoperative outcomes in RALR [33], and the authors were also unable to determine whether there was a robotic effect or whether the different study results could be attributed to inconsistency in the definitions of postoperative complications.

We rarely observed bile leakage or wound infections as postoperative complications and only non-surgical problems differed significantly between the four groups, which could also be related to the robotic approach. However, compared to Luberice et al., we did observe significant differences in the number of blood transfusions and the postoperative ICU stay within the four difficulty groups. Therefore, we conclude that the IWATE criteria are a good predictor of intraoperative surgical difficulty rather than of the postoperative outcome, which is a similar conclusion to that of Tanemura et al. in LLR [26].

In our cohort, RALR was found to be a safe and feasible surgical procedure. Our findings suggest that the use of RALR allows for performing difficult operations without worse postoperative outcomes [10,27]. Using RALR could offer a superior option when performing more complex operations compared to traditional laparoscopic approaches [31]. The improved ergonomic position, flexibility of laparoscopic instruments, three-dimensional view and greater range of motion allow for better accessibility to, e.g., the right superior posterior region and reduce surgical fatigue in long operations [32]. In addition, the reported case number required to comply with the learning curve in RALR is lower than that in LLR [10]. Nonetheless, it cannot be excluded that the surgeon’s experience in LLR might have an impact on the learning curve in RALR.

As shown, the IWATE criteria are helpful for predicting the intraoperative surgical difficulty in RALR. Moreover, the IWATE criteria may offer a helpful tool for selecting suitable operations according to the surgeon’s current skill level and may form a basis for a robotic training program in RALR for upcoming surgeons. Nevertheless, further development of the IWATE criteria could be necessary to ensure their applicability in RALR to predict perioperative outcomes. According to the recently published International Expert Consensus on robotic liver resections, a new DSS exclusively for RALR should be established [13].

As this was a retrospective study with a small number of patients, and one performed in a single center, our data have certain limitations. A larger number of patients underwent surgery, but not all of them met the inclusion criteria. In order to standardize the determination of the IWATE score, we included fewer patients, which resulted in a significantly more homogeneous patient cohort. With that said, the sample size of 58 patients is relatively large when considering that there are only a few centers that currently perform this procedure. Going forward, further studies are needed to evaluate the applicability of the IWATE criteria in RALR and to investigate whether or not there is a robotic effect.

## 5. Conclusions

In conclusion, we correlated the IWATE criteria as a DSS with the perioperative outcomes in RALRs. According to our results, the IWATE criteria can predict the intraoperative difficulty in RALR and could offer a basis for a robotic training program for surgeons in liver surgery. Whether or not there is a robotic effect must be clarified in further studies. Furthermore, a difficulty scoring system should be developed specifically for robotic resections.

## Figures and Tables

**Table 1:** Clinical characteristics of patients.

	Low	Intermediate	Advanced	Expert	Total	*p*-Value
Patients	9 (15.5)	31 (53.4)	11 (19.0)	7 (12.1)	58 (100)	
Age, years ^a^	63 (40–72)	56 (21–84)	50 (31–71)	55 (33–82)	56 (21–84)	0.718 ^b^
Sex						0.860 ^c^
Male	4 (44.4)	16 (51.6)	4 (36.4)	4 (57.1)	28 (48.3)	
Female	5 (55.6)	15 (48.4)	7 (63.6)	3 (42.9)	30 (51.7)	
BMI, kg/m^2 a^	26 (20–34)	25 (18–43)	27 (17–43)	28 (24–39)	25 (17–43)	0.173 ^b^
ASA classification						0.475 ^c^
1	1 (11.1)	4 (12.9)	0 (0)	0 (0)	5 (8.6)	
2	5 (55.6)	18 (58.1)	10 (90.9)	4 (57.1)	37 (63.8)	
3	3 (33.3)	9 (29.0)	1 (9.1)	3 (42.9)	16 (27.6)	
Diabetes	1 (11.1)	2 (6.5)	1 (9.1)	1 (14.3)	5 (8.6)	0.752 ^c^
Pulmonary	2 (22.2)	6 (19.4)	1 (9.1)	1 (14.3)	10 (17.2)	0.906 ^c^
Cardiac	1 (11.1)	4 (12.9)	1 (9.1)	3 (42.9)	9 (15.5)	0.243 ^c^
Renal insufficiency	1 (11.1)	1 (3.2)	0 (0)	0 (0)	2 (3.4)	0.512 ^c^
Arterial hypertension	5 (55.6)	11 (35.5)	4 (36.4)	2 (28.6)	22 (37.9)	0.725 ^c^
Liver cirrhosis	2 (22.2)	5 (16.1)	0 (0)	0 (0)	7 (12.1)	0.331 ^c^
Hepatitis B	1 (11.1)	1 (3.2)	0 (0)	1 (14.3)	3 (5.2)	0.271 ^c^
Hepatitis C	1 (11.1)	2 (6.5)	0 (0)	0 (0)	3 (5.2)	0.689 ^c^
Steatosis	1 (11.1)	1 (3.2)	1 (9.1)	0 (0)	3 (5.2)	0.447 ^c^
NASH	0 (0)	2 (6.5)	0 (0)	0 (0)	2 (3.4)	1.000 ^c^
History of smoking	2 (22.2)	16 (51.6)	5 (45.5)	4 (57.1)	27 (46.6)	0.442 ^c^
Alcohol abuse	1 (11.1)	1 (3.2)	0 (0)	0 (0)	2 (3.4)	0.512 ^c^
Extrahep. metastasis	1 (11.1)	4 (12.9)	0 (0)	0 (0)	5 (8.6)	0.868 ^c^
Prev. chemotherapy	1 (11.1)	7 (22.6)	1 (9.1)	2 (28.6)	11 (19.0)	0.703 ^c^
Prev. abdominal surgery	9 (100)	17 (54.8)	4 (36.4)	5 (71.4)	35 (60.3)	0.016 ^c^
Preop. bilirubin, mg/dL ^a^	0.5 (0.2–1.3)	0.4 (0.1–1.0)	0.3 (0.2–0.7)	0.5 (0.3–1.0)	0.4 (0.1–1.3)	0.108 ^b^
Preop. INR ^a^	1.0 (0.8–1.4)	1.0 (0.8–1.3)	1.0 (0.9–1.1)	1.0 (0.9–1.6)	1.0 (0.8–1.6)	0.515 ^b^
Preop. albumin ^d^, g/dL ^a^	4.1 (3.8–4.9)	4.5 (3.6–5.1)	4.4 (4.1–4.8)	4.4 (4.4–5.1)	4.5 (3.6–5.1)	0.904 ^b^

Values in parentheses are in percentages. ^a^ Values are median and range. ^b^ Kruskal–Wallis test. ^c^ Fisher’s exact test. ^d^ Serum albumin. BMI, body mass index; ASA, American Society of Anesthesiologists classification; NASH, nonalcoholic steatohepatitis; Extrahep., extrahepatic; Prev., previous; CTX, chemotherapy; Preop., preoperative; INR, international normalized ratio.

**Table 2:** Indications for robotic-assisted liver resection.

Indication	Low	Intermediate	Advanced	Expert	Total
PHB malignancy	4 (44.4)	8 (25.8)	4 (36.4)	3 (42.9)	19 (32.8)
HCC	2 (22.2)	5 (16.1)	3 (27.3)	1 (14.3)	11 (19.0)
ICC	1 (11.1)	3 (9.7)	1 (9.1)	2 (28.6)	7 (12.1)
GBC	1 (11.1)	0 (0)	0 (0)	0 (0)	1 (1.7)
Metastatic disease	3 (33.3)	11 (35.5)	2 (18.2)	2 (28.6)	18 (31.0)
CRLM	1 (11.1)	9 (29.0)	1 (9.1)	1 (14.3)	12 (20.7)
Other	2 (22.2)	2 (6.5)	1 (9.1)	1 (14.3)	6 (10.3)
Benign	2 (22.2)	12 (38.7)	5 (45.5)	2 (28.6)	21 (36.2)
FNH	1 (11.1)	6 (19.5)	1 (9.1)	0 (0)	8 (13.8)
Hepatic adenoma	0 (0)	1 (3.2)	1 (9.1)	0 (0)	2 (3.4)
Cyst	1 (11.1)	1 (3.2)	0 (0)	0 (0)	2 (3.4)
Hemangioma	0 (0)	4 (12.9)	1 (9.1)	2 (28.6)	7 (12.1)
Echinococcus cyst	0 (0)	0 (0)	2 (18.2)	0 (0)	2 (3.4)

Values in parentheses are in percentages. Fisher’s exact test showed a *p*-value of 0.840 between the IWATE criteria and indications. PHB, pancreatic hepatobiliary; HCC, hepatocellular carcinoma; ICC, intrahepatic cholangiocarcinoma; GBC, gallbladder carcinoma; CRLM, colorectal liver metastasis; FNH, focal nodular hyperplasia.

**Table 3:** Grading of patients according to the IWATE criteria and their six preoperative characteristics.

	Low	Intermediate	Advanced	Expert	Total	*p*-Value
Tumor location, segment						<0.001
S I	0 (0)	0 (0)	0 (0)	0 (0)	0 (0)	
S II	2 (22.2)	7 (22.6)	1 (9.1)	0 (0)	10 (17.2)	
S III	0 (0)	11 (35.5)	0 (0)	0 (0)	11 (19.0)	
S IVa	0 (0)	0 (0)	0 (0)	0 (0)	0 (0)	
S IVb	2 (22.2)	1 (3.2)	2 (18.2)	0 (0)	5 (8.6)	
S V	1 (11.1)	2 (6.5)	2 (18.2)	0 (0)	5 (8.6)	
S VI	4 (44.4)	3 (9.7)	2 (18.2)	0 (0)	9 (15.5)	
S VII	0 (0)	3 (9.7)	3 (27.3)	4 (57.1)	10 (17.2)	
S VIII	0 (0)	4 (12.9)	1 (9.1)	3 (42.9)	8 (13.8)	
Tumor size						<0.001 ^a^
≤30 mm	8 (88.9)	14 (41.9)	1 (9.1)	0 (0)	22 (37.9)	
≥30 mm	1 (11.1)	18 (58.1)	10 (90.9)	7 (100)	36 (62.1)	
Child–Pugh Score						1.000 ^a^
A or less	9 (100)	30 (96.8)	11 (100)	7 (100)	57 (98.3)	
B	0 (0)	1 (3.2)	0 (0)	0 (0)	1 (1.7)	
C	0 (0)	0 (0)	0 (0)	0 (0)	0 (0)	
Extent of liver resection						<0.001 ^a^
Partial resection	9 (100)	11 (35.5)	3 (27.3)	0 (0)	23 (39.7)	
Left lateral sectionectomy	0 (0)	17 (54.8)	1 (9.1)	0 (0)	18 (31.0)	
Segmentectomy	0 (0)	3 (9.7)	6 (54.5)	2 (28.6)	11 (19.0)	
Sectionectomy or more	0 (0)	0 (0)	1 (9.1)	5 (71.4)	6 (10.3)	
Proximity to major vessels	2 (22.2)	18 (58.1)	9 (81.8)	6 (85.7)	35 (60.3)	0.024 ^a^
HALS/Hybrid	0 (0)	0 (0)	0 (0)	0 (0)	0 (0)	

Values in parentheses are in percentages. ^a^ Fisher’s exact test. S., segment; HALS, hand-assisted laparoscopic surgery.

**Table 4:** Operative characteristics and perioperative outcome.

	Low	Intermediate	Advanced	Expert	Total	*p*-Value
Surgery time, min. ^a^	176 (121–244)	215 (37–410)	266 (199–410)	492 (231–671)	237 (37–671)	<0.001 ^c^
Con. procedure	2 (22.2)	13 (41.9)	2 (18.2)	5 (71.4)	29 (50.0)	0.027 ^d^
Conversion to open	0 (0)	0 (0)	1 (9.1)	1 (14.3)	2 (3.4)	0.152 ^d^
Pringle maneuver	0 (0)	1 (3.2)	2 (18.2)	3 (42.9)	6 (10.3)	0.016 ^d^
Pringle time, min. ^b^	0 (0 ± 0)	1 (1 ± 6)	6 (6 ± 13)	20 (20 ± 32)	4 (4 ± 14)	0.010 ^c^
ICU stay	0 (0)	3 (9.7)	3 (27.3)	6 (85.7)	12 (20.7)	<0.001 ^d^
ICU stay, days ^a^	0 (0 ± 0)	0 (0 ± 0.5)	1 (1 ± 1.4)	4 (4 ± 4.3)	1 (1 ± 1.9)	<0.001 ^c^
Reoperation	0 (0)	0 (0)	0 (0)	1 (14.3)	1 (1.7)	0.121 ^d^
PBT	0 (0)	0 (0)	1 (9.1)	2 (28.6)	3 (5.2)	0.023 ^d^
Length of stay, days ^a^	6 (3–12)	6 (3–15)	5 (3–21)	7 (4–37)	6 (3–37)	0.192 ^c^
R status						0.121 ^d^
R0	9 (100)	31 (100)	11 (100)	6 (85.7)	57 (98.3)	
R1	0 (0)	0 (0)	0 (0)	1 (14.3)	1 (1.7)	
Clavien–Dindo Grade						0.014 ^d^
0	4 (44.4)	23 (74.2)	9 (81.8)	2 (28.6)	38 (65.5)	
1	4 (44.4)	2 (6.5)	0 (0)	2 (28.6)	8 (13.8)	
2	1 (11.1)	4 (12.9)	1 (9.1)	0 (0)	7 (12.1)	
3a	0 (0)	2 (6.5)	1 (9.1)	1 (14.3)	3 (5.2)	
3b	0 (0)	0 (0)	0 (0)	2 (28.6)	2 (3.4)	
CCI ^b^	8 (8 ± 9)	6 (6 ± 11)	6 (6 ± 16)	23 (23 ± 24)	8 (8± 4)	0.052 ^c^
Complication rate						0.018 ^d^
None	4 (44.4)	23 (74.2)	9 (81.8)	2 (28.6)	38 (65.5)	
Mild	5 (55.6)	8 (25.8)	2 (1.2)	3 (42.9)	18 (31.0)	
Relevant	0 (0)	0 (0)	0 (0)	2 (28.6)	2 (3.4)	
Bile leakage	1 (11.1)	0 (0)	1 (9.1)	1 (14.3)	3 (5.2)	0.116 ^d^
Bleeding	0 (0)	0 (0)	1 (9.1)	1 (14.3)	2 (3.4)	0.152 ^d^
Wound infection	0 (0)	1 (3.2)	0 (0)	1 (14.3)	2 (3.4)	0.344 ^d^
Postop. hematoma	0 (0)	1 (3.2)	0 (0)	0 (0)	1 (1.7)	1.000 ^d^
Non-surg. problems	0 (0)	0 (0)	0 (0)	2 (28.6)	2 (3.4)	0.013 ^d^
Pulmonary comp.	2 (22.2)	3 (9.7)	1 (9.1)	3 (42.9)	9 (15.5)	0.143 ^d^
Cardiac comp.	1 (11.1)	0 (0)	0 (0)	1 (14.3)	2 (3.4)	0.106 ^d^
Urinary comp.	1 (11.1)	2 (6.5)	0 (0)	1 (14.3)	4 (6.9)	0.447 ^d^
Readmission	1 (11.1)	1 (3.2)	2 (18.2)	0 (0)	4 (6.9)	0.227 ^d^
Mortality	0 (0)	0 (0)	0 (0)	0 (0)	0 (0)	

Values in parentheses are in percentages. ^a^ Values are median and range. ^b^ Values are mean and standard deviation. ^c^ Kruskal–Wallis test. ^d^ Fisher’s exact test. Min., minutes; Con. Procedure, concomitant procedure; ICU, intensive care unit; PBT, perioperative blood transfusion; CCI, comprehensive complication index; Postop., postoperative; Non-surg., non-surgical.

## Data Availability

The data presented in this study are available on request from the corresponding author. The data are not publicly available.
